# Assessing Trends in Hospitalizations for Breast Cancer among Women in Korea: A Utilization of the Korea National Hospital Discharge In-depth Injury Survey (2006–2020)

**DOI:** 10.1007/s44197-024-00229-1

**Published:** 2024-04-29

**Authors:** Jieun Hwang, Jeong-Hoon Jang

**Affiliations:** 1 Department of Health Administration, College of Health Science, Dankook University, 31116 Cheonan City, Chungcheongnam-do South Korea; 2 College of Pharmacy, Daegu Catholic University, 38430 Gyeongsan-si, Gyeongsangbuk-do South Korea

**Keywords:** Breast cancer, Patients, Discharge, Comorbidity

## Abstract

**Objective:**

Breast cancer poses a significant health threat globally and particularly in Korea, where mortality rates have risen notably. In this study, we analyzed the characteristics of breast cancer patients discharged in Korea over the past 15 years and explored the association between comorbidities and treatment outcomes to propose effective strategies for managing cancer patients. Understanding these dynamics is vital for informing tailored management strategies and optimizing healthcare system sustainability.

**Methods:**

This study utilized cross-sectional data from the Korea National Hospital Discharge In-depth Injury Survey from 2006 to 2020. Each year, among patients discharged from hospital with 100 beds or more, those identified with breast cancer patients were based on their primary diagnosis code (C50) according to the ICD-10, as recorded in their medical records.

**Results:**

Between 2006 and 2020, an estimated 499,281 breast cancer patients were discharged, with an average annual percent change (AAPC) of 5.2% (95% CI 4.2–6.2, *p* <.05). A notable increase in AAPC was particularly evident among those aged 60 years and old. Across all age groups, there was a consistent increasing trend in the risk of mortality as the CCI score increased (*p* <.05). The risk of comorbidity was more pronounced in younger age groups compared to older age groups.

**Conclusions:**

The increasing life expectancy is expected to lead to a continued rise in the number of elderly breast cancer patients. Countermeasures are needed to address this trend through appropriate diagnosis and treatment planning. Particularly, considering comorbidities in breast cancer treatment plans is necessary to promote positive treatment outcomes, especially in younger breast cancer patients.

## Background

Breast cancer, a prevalent neoplastic disease presenting a substantial health concern for women globally, manifests as a malignant neoplasm in the breast cells [1, 2]. The global incidence of breast cancer continues to surge, driven by fasctors such as population aging, shifts in demographic structure, and evolving lifestyles [1]. Moreover, recent advancements in medical technology have facilitated more accurate and early diagnosis, leading to the detection of an increasing number of breast cancer cases [3].

In Korea, breast cancer is recognized as a significant public health concern, posing a threat to the health of women as well [4]. In 2021, leading causes of death among Korean women included malignant neoplasms (cancer), heart disease, cerebrovascular disease, pneumonia, and Alzheimer’s disease, in that order. Analyzing cancer mortality rates, lung cancer ranked highest followed by colorectal cancer, pancreatic cancer, liver cancer, breast cancer, and stomach cancer [5]. Notably, the mortality rate of breast cancer has increased by 36% compared to a decade ago [6].

Breast cancer is a complex disease resulting in the formation of a malignant tumor in the breast cells [1, 2]. Individuals with a strong genetic predisposition to breast cancer often harbor genetic variations, notably BRCA1 and BRCA2, which elevate the risk of developing breast cancer [7]. Furthermore, alterations in the levels of, including estrogen and progesterone, play a role in the onset of breast cancer hormones [8]. Beyond genetic and hormonal factors, a woman’s personal history of reproductive events such as menarche, pregnancy, and breastfeeding, alongside lifestyle habits including smoking, alcohol consumption, exercise patterns, and dietary choices, are significantly correlated with the occurrence of breast cancer [1, 2]. Understanding this intricate interplay of factors is crucial for comprehensive approaches to prevention and early detection in the management of breast cancer.

Nevertheless, there remains a gap in understanding the discharge trends and outcomes for breast cancer patients, particularly among Korean women. The differences in demographic structures and healthcare services across different countries can contribute to notable variations in treatment outcomes. Comorbidities not only shape a patient’s treatment approach and methods but also significantly impact discharge results, hospital stays, and overall hospital management aspects [9, 10]. As breast cancer development involves intricate interactions of various factors, an examination of a patient’s comorbidities becomes essential, influencing both treatment strategies and prognoses.

In our current study, we aimed to analyze the scale and characteristics of discharged breast cancer patients in Korea over the past 15 years, utilizing patient discharge information. We also extended to our analysis to evaluate the association between comorbidities and the treatment outcomes of Korean breast cancer patients. Exploring this intricate aspect, we sought to unravel the intricate relationship between comorbidities and breast cancer treatment, contributing valuable insights that can inform the development of effective management methods. This approach may take into account not only the immediate well-being of the patient but also considers the long-term quality of life and the sustainability of healthcare systems.

## Materials and Methods

### Study Population

This study utilized secondary data from the Korea National Hospital Discharge In-depth Injury Survey (KNHDIS) conducted between 2006 and 2020. The survey, conducted annually by the Korea Disease Control and Prevention Agency (KDCA) since 2005, aims to inform the development of cost-effective health policies by capturing the scale and characteristics of discharged patients [11].

The survey targets patients discharged from general hospitals with 100 beds or more nationwide. Based on the hospital size, 170 hospitals are selected as sample hospitals, and approximately 9% of samples are chosen, collecting demographic information, admission details, disease, and treatment information, as well as injury data of discharged patients from medical records [11].

**Fig. 1:**
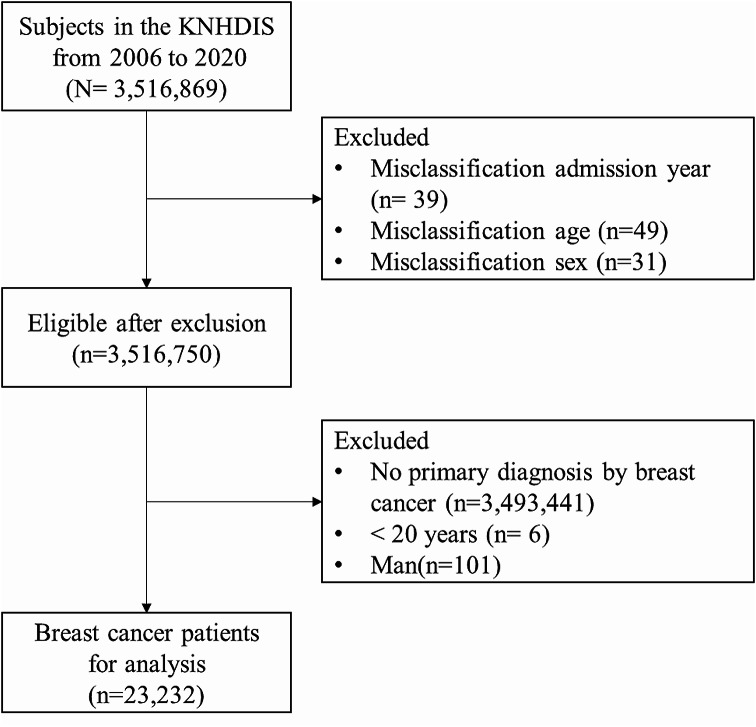
Flow chart by study population. KNHDIS; Korea National Hospital Discharge In-depth Injury Surve

The study population procedure was carried out as shown in Fig. 1. A total of 3,516,869 subjects were participated between 2006 and 2020. We excluded a few cases from the analysis due to misclassified data (*n* = 88).

The survey contains one primary diagnosis and 20 secondary diagnoses. In this study, individuals discharged with the primary diagnosis coded as C50 (Malignant neoplasm of the breast) according to ICD-10 were categorized as breast cancer patients. We excluded cases without a primary diagnosis of breast cancer (*n* = 3,493,441). The final study selected 23,232 (weighted *N* = 499,281) breast cancer patients as our study subjects, excluding those under 20 years old (*n* = 6) and men (*n* = 101) from the study population.

### Variables

The variables utilized in this study included demographic information of patients, categorized by age group (< 40, 40–59, ≥ 60 years). Additionally, this considered admission route(Emergency department, Outpatient department, Others), insurance type(National Health Insurance, Medicaid, Others), number of hospital beds(100–299, 300–499, 500–999, ≥ 1,000 beds), operation history(No, Yes), and treatment outcome(Alive, Death). Treatment outcome was categorized based on the patient’s conditions at the time of discharge. Cases where patients died at the time of discharged were classified as “Death”, while all other discharges, such as to home or transfer, were classified as “Alive”.

In this study, secondary diagnoses accompanying the primary diagnosis C50 at discharge were considered as comorbidities. These comorbidities influencing treatment outcomes, specifically mortality, among breast cancer patients were restricted those outlined in the Charlson Comorbidity Index (CCI) [12]. CCI is an index assigns scores to comorbidities based on the relative risk of 19 major diseases, including ischemic heart disease, diabetes mellitus, hypertension, and others. Typically, CCI categorizes comorbidities into scores of 0, 1, 2 or 3+.

### Research Model and Statistical Analyses

We examined the trends of breast cancer patients by age group each year. As outlined in the data resource profile, the survey implemented a 2-stage stratified cluster sampling scheme. In the first stage, individual hospitals were designated as primary sampling units, and in the second stage, discharged cases from the sampled hospitals were selected as secondary sampling units. The data utilizes a two-stage stratified cluster sampling methodology, which requires the application of complex sampling techniques. We calculated weighted totals of discharged patients for each year accordingly. Subsequently, based on the 2015 population, we calculated the age-standardized discharge rate and presented the average annual percent change(AAPC).

To identify characteristics of discharged breast cancer patients, we conducted a frequency analysis of admission route, insurance type, treatment outcomes, number of hospital beds, and operation history information based on age groups (< 40, 40–59, 60+) and tested chi-square analysis for statistical significance of group differences. Also, CCI used frequency analysis based on age groups and conducted chi-square to test the statistical significance of differences among these groups.

Subsequently, to compare the association between treatment outcomes (mortality) among breast cancer patients with CCI comorbidities in each group, multiple logistic regression analysis was analyzed resulting in the calculation of adjusted odds ratio(aOR) and 95% confidence intervals (CI). In analysis, demographic characteristics (admission route, insurance type, hospital beds, operation) were used as covariates for adjustment. All analysis was conducted using SAS software (version 9.4, SAS Institute, Cary, NC, US). We considered the results statistically significant when the p-value was less than the significance level of 0.05 (*p* <.05).

## Results

### The Number of Discharged Breast Cancer Patients over the Past 15 Years

**Fig. 2:**
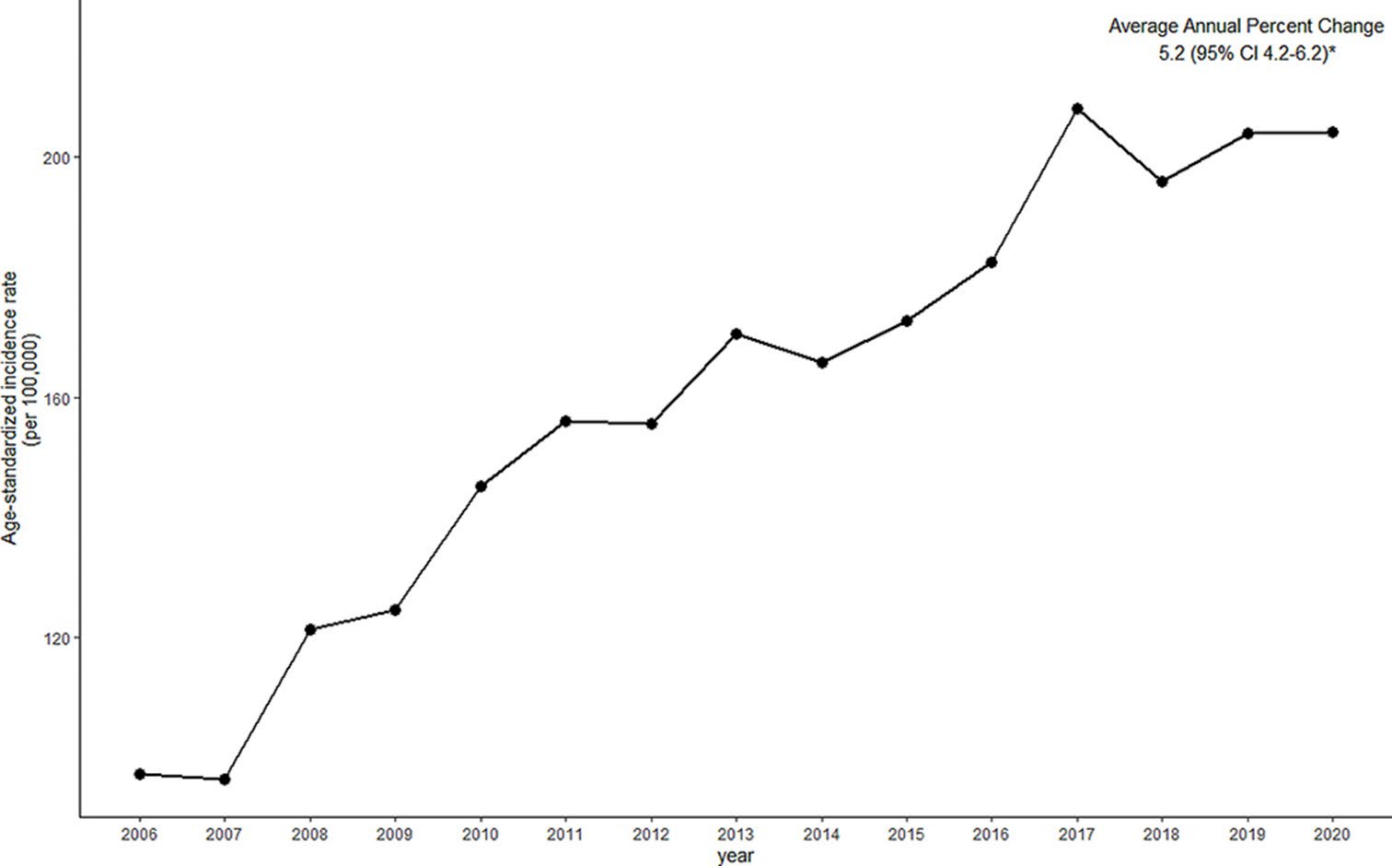
Trends in the discharge rate of breast cancer patients between 2006 and 2020 in Korea. *p <.05

We estimated that 499,281 patients were discharged after hospitalization treatment for breast cancer from 2006 to 2020. The age-standardized incidence rate showed a trend of increasing and decreasing, while AAPC was 5.2% (95% CI 4.2–6.2, *p* <.05) which showed overall increasing trend (Fig. 2).

**Fig. 3:**
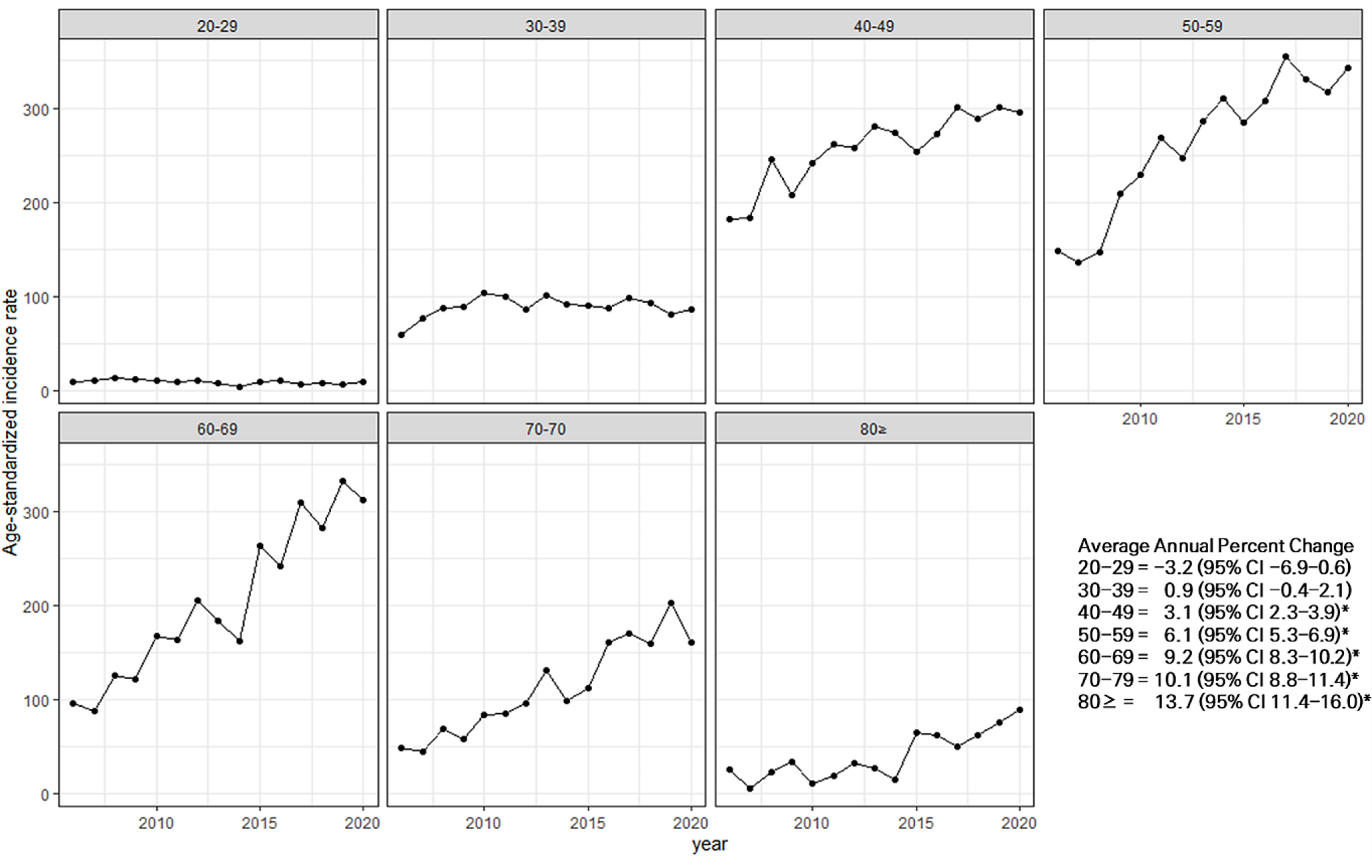
Trends in age-standardized incidence rate and average annual percent change by age group. *p <.05

By age groups, AAPC was highest in the 80 years and older age group at 13.7% (95% CI 11.4–16.0, *p* <.05), followed by the 70–79 years group (AAPC = 10.1, 95% CI 8.8–11.4, *p* <.05), 60–69 years group (AAPC = 9.2, 95% CI 8.3–10.2, *p* <.05), 50–59 years group (AAPC = 6.1, 95% CI 5.3–6.9, *p* <.05), and 40–49 years group (AAPC = 3.1, 95% CI 2.3–3.9, *p* <.05) (Fig. 3).

### Demographic Characteristics of Discharged Breast Cancer Patients

By age group, outpatient admissions were more common than emergency or other types of admissions in all age groups, but the proportion of emergency admissions was highest in those aged 60 and older (*p* <.05) (Table 1). The type of insurance also showed that health insurance was more common than medical aid or other types, with the highest proportion of medical aid coverage in the group aged 60 and older (*p* <.05). In terms of hospital size, the 500–999 bed category had the highest frequency across all age groups, but as age increased, the proportion of 500–999 beds increased, while the proportion of over 1,000 beds decreased (*p* <.05). The percentage of patients with an operation history was higher than those without across age groups, but the proportion of surgical history in the 40–59 age group was lower compared to other age groups (*p* <.05). The proportion of deceased patients tended to increase with age (*p* <.05).

**Table 1:** Comparative analysis of sociodemographic characteristics among discharged breast cancer patient by age groups

	Total (*n* = 499,281)		< 40 years (*n* = 221,636)		40–59 years (*n* = 211,017)		60 + years (*n* = 66,628)		p-value^†^
	weighted n	weighted %	weighted n	weighted %	weighted n	weighted %	weighted n	weighted %	
Admission route									0.0006
Emergency department	51,717	10.4	20,752	9.4	23,502	11.1	7,463	11.2	
Outpatient department	445,493	89.2	199,808	90.2	186,661	88.5	59,024	88.6	
Others	2,071	0.4	1,076	0.5	854	0.4	141	0.2	
Insurance type									< 0.0001
NHI	463,357	92.8	208,589	94.1	196,051	92.9	58,717	88.1	
Medicaid	32,239	6.5	11,096	5.0	13,498	6.4	7,645	11.5	
Others	3,685	0.7	1,951	0.9	1,468	0.7	266	0.4	
Number of hospital beds						-		-	< 0.0001
100–299	56,364	11.3	25,183	11.4	23,742	11.3	7,439	11.2	
300–499	46,383	9.3	19,600	8.8	19,178	9.1	7,605	11.4	
500–999	244,960	49.1	104,193	47.0	106,404	50.4	34,363	51.6	
≥1000	151,574	30.4	72,660	32.8	61,693	29.2	17,221	25.8	
Operation history		0.0				-		-	< 0.0001
No	226,133	45.3	95,895	43.3	100,884	47.8	29,354	44.1	
Yes	273,148	54.7	125,741	56.7	110,133	52.2	37,274	55.9	
Treatment outcome		0.0				-		-	< 0.0001
Alive	481,931	96.5	215,492	97.2	203,014	96.2	63,425	95.2	
Death	17,350	3.5	6,144	2.8	8,003	3.8	3,203	4.8	

^†^Rao-Scott Chi-Square test

NHI, National Health Insurance

### Comorbidity (CCI) among Discharged Breast Cancer Patients

In all age groups, metastatic carcinoma was the most common comorbidity (under 40 years 32.9%, 40–59 years 33.0%, 60 years and above 31.8%) (Table 2). Additionally, in the group aged under 40, cases with cancers other than breast cancer accounted for 3.8%, and those with diabetes without complications were 1.6%. For the 40–59 age group, cases with diabetes without complications (5.4%) were more prevalent than cases with other cancers (4.1%). Similarly, in the 60 and above age group, cases with diabetes without complications (12.0%) outnumbered cases with other cancers (5.4%). Although the majority of individuals in each age group had a CCI score of 0, those with a CCI score of 3 or higher were most prevalent in the 60 and above age group (12.8%), followed by the 40–59 age group (10.9%), and those under 40 years old (8.9%).

**Table 2:** Comparative analysis of comorbidity distribution among discharged breast cancer patients by age groups

CCI	< 40 years (*n* = 221,636)		40–59 years (*n* = 211,017)		60 + years (*n* = 66,628)		p-value^†^
	weighted n	weighted %	weighted n	weighted %	weighted n	weighted %	
Myocardial infarction	-	-	93	0.0	113	0.2	-
Congestive heart failure	168	0.1	622	0.3	398	0.6	< 0.0001
Peripheral vascular disease	90	0.0	54	0.0	-	-	-
Cerebrovascular accident/transient ischemic attacks	214	0.1	920	0.4	477	0.7	< 0.0001-
Dementia	-	-	50	0.0	471	0.7	
COPD	598	0.3	891	0.4	767	1.2	< 0.0001
Connective tissue disease	278	0.1	314	0.1	220	0.3	0.0888
Peptic ulcer disease	-	-	-	-	-	-	-
Liver disease	1,716	0.8	2,194	1.0	818	1.2	0.0898
Diabetes without complications	3,470	1.6	11,402	5.4	7,990	12.0	< 0.0001
Diabetes with complications	70	0.0	190	0.1	729	1.1	< 0.0001
Paraplegia and hemiplegia	301	0.1	303	0.1	84	0.1	0.9759
Renal disease	573	0.3	718	0.3	672	1.0	< 0.0001
Cancer	8,437	3.8	8,709	4.1	3,598	5.4	0.0051
Moderate or severe liver disease	255	0.1	520	0.2	28	0.0	0.0082
Metastatic carcinoma	72,819	32.9	69,689	33.0	21,161	31.8	0.5307
AIDS/HIV	20	0.0	-	-	-	-	-
CCI score 3	19,754	8.9	23,091	10.9	8,559	12.8	< 0.0001
CCI score 2	10,446	4.7	12,480	5.9	4,710	7.1	
CCI score 1	50,461	22.8	47,976	22.7	16,468	24.7	
CCI score 0	140,975	63.6	127,470	60.4	36,891	55.4	

^†^Rao-Scott Chi-Square test

CCI, Charlson Comorbidity Index; COPD, Chronic Obstructive Pulmonary Disease; AIDS, Acquired Immunodeficiency Syndrome; HIV, Human Immunodeficiency Virus

### The Association between Comorbidities in Breast Cancer Patients (Primary Diagnosis) and Treatment Outcomes (Death)

In the age group under 40, moderate or severe liver diseases and metastatic cancer were associated with mortality among discharged breast cancer patients (Table 3). Discharged breast cancer patients who were accompanied by moderate or severe liver diseases had a 56.0 times (95% CI 12.7–247.0) higher risk of mortality. Furthermore, patients with metastatic carcinoma had a 8.6 times (95% CI 5.7–12.9) higher risk of mortality. The risk of mortality showed steadily increase as the CCI score rose(CCI score 3 aOR = 19.0, 95% CI 11.6–31.1; CCI score 2 aOR = 14.9, 95% CI 8.3–26.8; CCI score aOR = 3.8, 95% CI 2.2–6.6).

**Table 3:** Association between comorbidities in discharged breast cancer patient and treatment outcome (death)

CCI	< 40 years		40–59 years		60 + years	
	aOR	(95% CI)	aOR	(95% CI)	aOR	(95% CI)
Myocardial infarction	-		-		3.7	(0.2–56.1)
Congestive heart failure	2.0	(0.1–26.7)	5.9	(1.5–22.9) ^*^	4.4	(0.9–19.8)
Peripheral vascular disease	-		-		-	
Cerebrovascular accident/ transient ischemic attacks	1.6	(0.1–18.7)	3.8	(1.7–8.7) ^*^	2.3	(0.5–10.9)
Dementia	0.1	(0.1–0.1)	22.8	(2.1-247.1) ^*^	4.3	(0.6–29.4)
COPD	3.9	(0.5–32.1)	1.2	(0.2–6.7)	0.6	(0.1–3.2)
Connective tissue disease	1.2	(0.1–13.2)	10.1	(2.8–36.0) ^*^	7.3	(0.9–59.2)
Peptic ulcer disease	-		-		-	
Liver disease	2.0	(0.4–8.7)	1.6	(0.6–4.1)	1.3	(0.3–5.6)
Diabetes without complications	2.3	(0.9–5.6)	0.9	(0.5–1.4)	1.3	(0.8–2.2)
Diabetes with complications	-		10.4	(0.9-116.2)	1.6	(0.3–9.2)
Paraplegia and hemiplegia	1.8	(0.3–11.8)	2.0	(0.2–23.6)	1.3	(0.1–25.5)
Renal disease	1.0	(0.1–7.3)	5.8	(2.3–14.4) ^*^	2.4	(0.5–11.4)
Cancer	1.1	(0.5–2.3)	1.4	(0.7–2.6)	0.9	(0.3–2.6)
Moderate or severe liver disease	56.0	(12.7–247.0) ^*^	38.2	(12.5-116.6) ^*^	3.9	(0.2–72.8)
Metastatic carcinoma	8.6	(5.7–12.9) ^*^	8.4	(5.7–12.2) ^*^	4.9	(2.9–8.1) ^*^
AIDS/HIV	-		-		-	
CCI score 3 (ref.=0)	19.0	(11.6–31.1) ^*^	17.2	(11.0-26.8) ^*^	6.8	(3.7–12.6) ^*^
CCI score 2 (ref.=0)	14.9	(8.3–26.8) ^*^	11.1	(6.8–18.0) ^*^	5.5	(2.7–11.3) ^*^
CCI score 1 (ref.=0)	3.8	(2.2–6.6) ^*^	3.8	(2.3–6.2) ^*^	1.7	(0.9–3.2)

CCI, Charlson Comorbidity Index; aOR, adjusted Odds Ratio; CI, Confidence Interval; COPD, Chronic Obstructive Pulmonary Disease; AIDS, Acquired Immunodeficiency Syndrome; HIV, Human Immunodeficiency Virus

*p <.05

In the age group 40–59, congestive heart failure(aOR = 5.9, 95% CI 1.5–22.9), cerebrovascular accident(aOR = 3.8, 95% CI 1.7–8.7), dementia(aOR = 22.8, 95% CI 2.1-247.1), connective tissue disease(aOR = 10.1, 95% CI 2.8–36.0), renal disease(aOR = 5.8, 95% CI 2.3–14.4), moderate or severe liver diseases(aOR = 38.2, 95% CI 12.5-116.6) and metastatic carcinoma(aOR = 8.4, 95% CI 5.7–12.2) were associated with mortality among discharged breast cancer patients. In this group as well, there was a continuous increase in the risk of mortality with the increase in CCI score(CCI score 3 aOR = 17.2, 95% CI 11.1–26.8; CCI score 2 aOR = 11.1, 95% CI 6.8–18.0; CCI score aOR = 3.8, 95% CI 2.3–6.2).

In the group aged 60 and above, metastatic carcinoma(aOR = 4.9, 95% CI 2.9–8.1) were associated with mortality of discharged breast cancer patients. In this group, the risk of mortality continued to increase with the rising CCI score(CCI score 3 aOR = 6.8, 95% CI 3.7–12.6; CCI score 2 aOR = 5.5, 95% CI 2.7–11.3).

## Discussion

The number of discharged breast cancer patients in Korea has been on a rising trend for the past 15 years. Especially in all age groups over 30, the number of breast cancer patients has been on the rise, with the highest AAPC observed among older age group women. In case of discharged breast cancer patients, there was a demonstrated increase in the association between a higher number of comorbidities and mortality. The extent of this association varied across different age groups.

This study’s observations indicate that the number of breast cancer patients in Korea is exhibiting an overall increase, aligning with a global trend. The global number of breast cancer patients was estimated to be 2.3 million in 2020, signifying an annual growth rate of approximately 0.33% since 1990 [13]. In the last 15 years, South Korea has experienced a substantial surge in breast cancer cases, demonstrating an average annual growth of 5.2%. This upward trajectory is particularly noteworthy among the elderly population, a phenomenon likely influenced by the country’s extended average life expectancy [14]. Indeed, breast cancer arises from various complex factors, with age being recognized as the most significant risk factor [15]. In the United Kingdom, breast cancer primarily affects women aged 70 and older, representing over one-third of total cases, while in developing countries, breast cancer in women under 50 constitutes 50% of the overall occurrences [14, 15]. The findings of this study emphasize the need for more proactive preventive efforts, as breast cancer incidence is projected to increase, ranging from a minimum of 32% in developed countries to as much as 95% in developing nations, due to globalization and economic growth leading to an increase in average life expectancy [14].

The CCI demonstrates an increasing risk based on the number of comorbidities and age. This trend not only affects the treatment outcomes presented in this study but also has implications for survival, highlighting the importance of managing patients with multiple health conditions [16]. In essence, the presence of comorbidities or complications in patients complicates breast cancer management, influencing treatment decisions and inevitably impacting treatment tolerance, leading to potential implications for treatment outcomes [16–18]. The findings of this study, particularly the heightened risk of mortality associated with an increasing CCI score in younger age groups, underscore the necessity for comprehensive comorbidity management in patients of lower age ranges.

In this context, CCI is increasingly being utilized as a tool to assess the health status of breast cancer patients, and it seems valuable in formulating treatment strategies [16–18]. By identifying coexisting medical conditions and predicting treatment outcomes, a more proactive treatment approach can be devised [16–18]. The results of this study suggest the need for a more meticulous treatment approach in breast cancer patients with comorbidities, emphasizing the importance of considering concurrent health conditions in treatment planning.

Especially in the treatment of breast cancer, decisions are often made based solely on the patient’s age, overlooking other factors such as comorbidities that can significantly impact the effectiveness or potential adverse effects of the treatment [19, 20]. While some guidelines suggest that there is no difference in treatment outcomes regardless of the presence of comorbidities when using the same treatment approach [21], the actual scientific evidence regarding the treatment effectiveness in breast cancer patients with moderate to severe comorbidities is lacking. As indicated in this study, the treatment outcomes vary based on what comorbidities were present and to what extent, underscoring the need to consider not only the patient’s age but also the accompanying conditions when formulating breast cancer treatment plans.

It is known that there is an association between breast cancer and liver disease [22, 23]. In particular, it is known that breast cancer patients diagnosed with nonalcoholic fatty liver disease (NAFLD) are more likely to exhibit a poor prognosis in terms of recurrence [24]. Metabolic syndrome, such as diabetes and obesity, is known to be more prevalent in breast cancer patients [25]. Similarly, liver diseases also encompass risk factors such as metabolic abnormalities and obesity, implying a mechanistic connection between breast cancer and liver diseases [26]. In this study, it also revealed that there was a close association between mortality in breast cancer discharged patients under the age of 60 and liver diseases, as well as metastatic cancer. The incidence rate of breast cancer in Korea is reported to be lower compared to Western populations [27, 28]. However, it is reported that the proportion of breast cancer patients in younger age groups is higher in Korea compared to Western populations and these patients exhibited rapid tumor progression and metastasis to distant organs [29, 30]. Therefore, it is essential to understand the mechanistic connections between co-existing conditions to elucidate effective treatment approaches against breast cancer.

The present study has significance in examining the long-term perspective of breast cancer discharged patients in Korean women and investigating the association between comorbidities in breast cancer patients and treatment outcomes. However, there are several limitations of the present study that should be noted. First, this study defined patients based solely on the primary diagnosis when estimating discharged patients per year. Therefore, even if there were secondary diagnoses with breast cancer codes, those patients were not considered as subjects of the study. As a result, the actual number of discharged breast cancer patients might have been underestimated. Also, the KNHDIS only surveys all acute general hospitals with 100 beds. It has limitations in identifying patients discharged from hospitals with fewer than 100 beds or specialized hospitals such as single specialized hospitals, nursing hospitals, geriatric hospitals, veterans’ hospitals, military hospitals, and rehabilitation hospitals. There is a need to consider conducting surveys that encompass all type of hospitals. Furthermore, KNHDIS does not collect personal identification information. All the data related to a particular discharge case originate from one hospital only, which may lead to difficulties in distinguishing cases of readmission to multiple hospitals and potential duplicates. Therefore, it will be necessary to implement strategies to address these issues in future research designs. Fourth, the analysis was conducted solely based on the presence of the disease and treatment outcomes for each patient, without considering other risk factors associated with breast cancer such as family history, smoking, alcohol consumption, physical activity, etc. Therefore, caution is needed when interpreting the results due to the omission of these relevant risk factors. Fifth, it’s challenging to present the casual relationship of treatment outcomes because KNHDIS is an analysis of cross-sectional data by year. Furthermore, since there are limitations in proving the temporal sequence of comorbidities, the presence of comorbidities implies an association rather than a casual relationship with treatment outcomes. Therefore, caution is needed when interpreting this aspect. However, it is necessary to establish the relationship between breast cancer and comorbidities presented in this study through mechanisms of subsequent disease occurrence in the future.

## Conclusion

This study aimed to analyze the scale and characteristics of breast cancer discharged patients and evaluate the association between comorbidities and their treatment outcomes using discharge patient information in Korea over the past 15 years. The research findings indicate a continuing upward trend in breast cancer discharged patients in Korea over the past 15 years, particularly showing an increasing trend across all age groups above 30 years, with the highest AAPC observed in older age group. When it comes to discharged breast cancer patients, there is a higher association between comorbidities and mortality, with varying impacts of comorbidities across different age groups. Considering the expected increase in breast cancer patients, especially with the aging population, it becomes crucial to formulate treatment plans that account for comorbidities to ensure positive treatment outcomes.

## Data Availability

The datasets analysed during the current study are available from the Korea Disease Control and Prevention Agency on reasonable request. [http://www.kdca.go.kr/contents.es? mid=a20303010502]

## Abbreviations

KDCA: Korea Disease Control and Prevention Agency
AAPC: average annual percent change
CCI: Charlson Comorbidity Index
aOR: adjusted odds ratio
CI: confidence intervals
NAFLD: nonalcoholic fatty liver disease
NHI: National Health Insurance
COPD: Chronic Obstructive Pulmonary Disease
AIDS: Acquired Immunodeficiency Syndrome
HIV: Human Immunodeficiency Virus
